# Supplementary figures and images for: Evaluation of ERIC-PCR and MALDI-TOF as typing tools for multidrug resistant Klebsiella pneumoniae clinical isolates from a tertiary care center in India
Source: PLoS One. 2022 Nov 17;17(11):e0271652. doi: 10.1371/journal.pone.0271652 (PMC9671336; doi:10.1371/journal.pone.0271652)

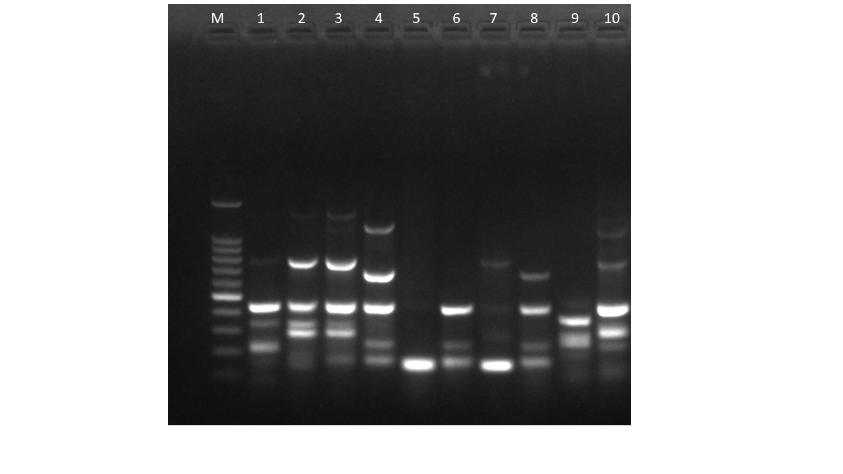

Supplement: S1 Fig — Lane M represents molecular marker(100bp), lane 1–10 represents samples showing characteristic ERIC banding pattern. (TIF) [file pone.0271652.s001.tif]
