# Supplementary material for: Evaluation of ERIC-PCR and MALDI-TOF as typing tools for multidrug resistant Klebsiella pneumoniae clinical isolates from a tertiary care center in India
Source: PLoS One. 2022 Nov 17;17(11):e0271652. doi: 10.1371/journal.pone.0271652 (PMC9671336; doi:10.1371/journal.pone.0271652)
Supplement: S1 Table — (DOCX) [file pone.0271652.s002.docx]

**S1 Table.** The list of primer sequences used for screening resistance genes.

| **Gene targeted** | **Primers** | **Product size (bp)** |
| --- | --- | --- |
|  |  |  |
| TEM | F:CATTTCCGTGTCGCCCTTATTC  R:CGTTCATCCATAGTTGCCTGAC | 800 |
| SHV | F:AGCCGCTTGAGCAATTAAAC  R:ATCCCGCAGATAAATCACCAC | 713 |
| OXA-1 | F:GGCACCAGATTCAACTTTCAAG  R:GACCCCAAGTTTCCTGTAAGTG | 564 |
| IMP | F:TTGACACTCCATTTACDG  R:GATYGAGAATTAAGCCACYCT | 139 |
| VIM | F:GATGGTGTTTGGTCGCATA  R:CGAATGCGCAGCACCAG | 390 |
| KPC | F:CATTCAAGGGCTTTCTTGCTGC  R:ACGACGGCATAGTCATTTGC | 538 |
| NDM-1 | F:GGTTTGGCGATCTGGTTTTC  R:CGGAATGGCTCATCACGATC | 621 |
| CTXM-15 | F:AGAATAAGGAATCCCATGGTT  R:ACCGTCGGTGACGATTTTAG | 913 |
| OXA-48 | F:TATATTGCATTAAGCAAGGG  R: CACACAAATACGCGCTAACC | 800 |
| MCR1 | F:CGGTCAGTCCGTTTGTTC  R :CTTGGTCGGTCTGTAGGG | 340 |
