# Supplementary material for: Evaluation of ERIC-PCR and MALDI-TOF as typing tools for multidrug resistant Klebsiella pneumoniae clinical isolates from a tertiary care center in India
Source: PLoS One. 2022 Nov 17;17(11):e0271652. doi: 10.1371/journal.pone.0271652 (PMC9671336; doi:10.1371/journal.pone.0271652)
Supplement: S2 Table — (DOCX) [file pone.0271652.s003.docx]

**S2 Table.** Detailed distribution of samples collected from different hospital wards/units as per their ERIC types(e1-e40).
